# Supplementary figures and images for: A novel mitovirus associated with the fungal entomopathogen Zoophthora radicans
Source: PLoS One. 2025 Sep 5;20(9):e0331239. doi: 10.1371/journal.pone.0331239 (PMC12412937; doi:10.1371/journal.pone.0331239)

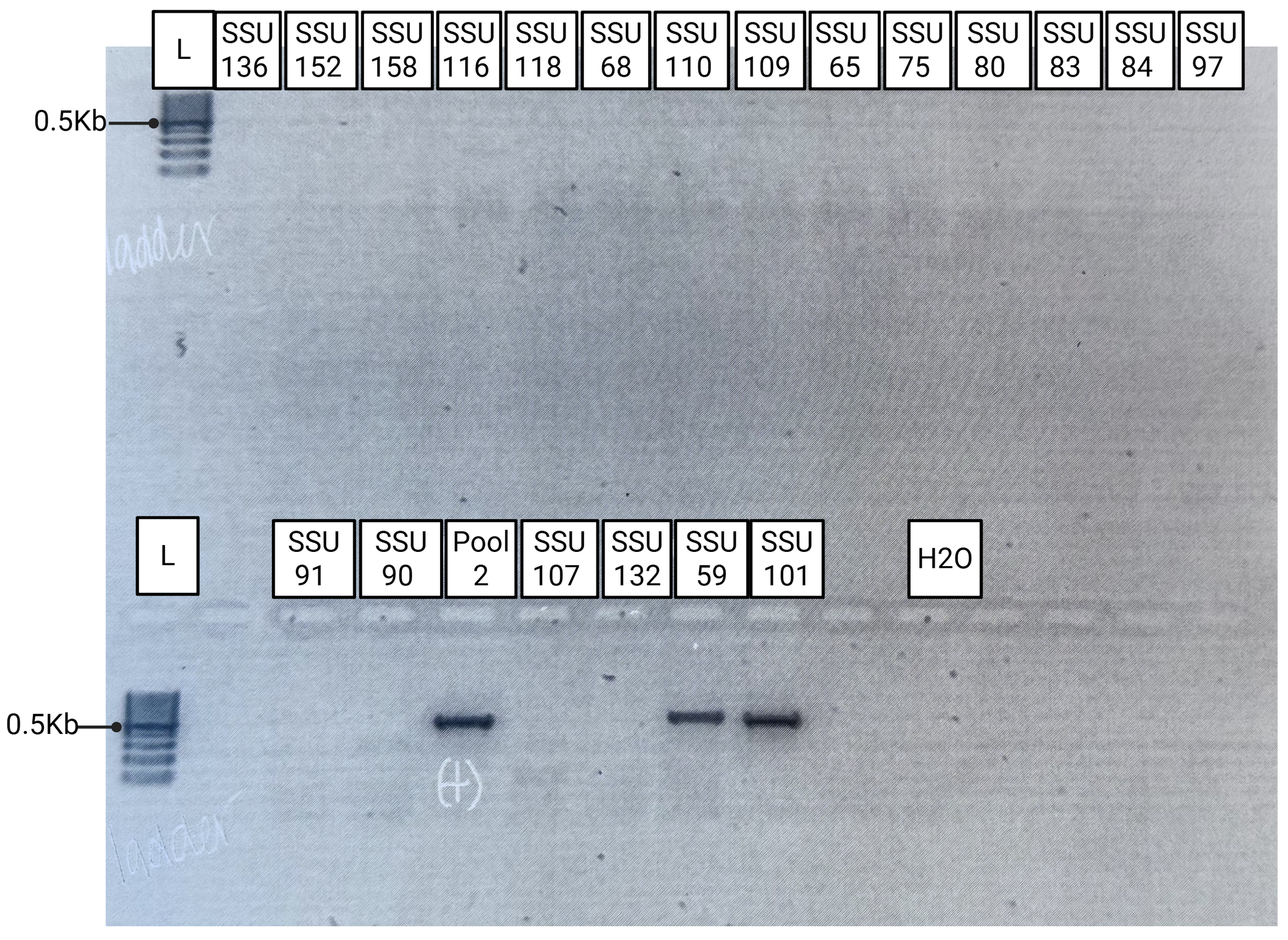

Supplement: S1 Fig — Gel image was taken via iPhone camera from UV transilluminator, inverted and labelled using Biorender. (TIF) [file pone.0331239.s002.tif]
