# Supplementary material for: A novel mitovirus associated with the fungal entomopathogen Zoophthora radicans
Source: PLoS One. 2025 Sep 5;20(9):e0331239. doi: 10.1371/journal.pone.0331239 (PMC12412937; doi:10.1371/journal.pone.0331239)
Supplement: Attachment — Submitted filename: Response to Reviewers.docx [file pone.0331239.s004.docx]

Reponses to reviewers for PLOS One

**Additional Editor Comments:** Please revise and improve as mentioned by reviewer #2 data availability, methods descriptions, and phylogenetic relationships, as their suggestions are relevant for reproducibility and transparency. I do not expect a virus isolation experiment, just keep in mind the comments to revise in the discussion the limitations for host assignment.

Thanks to the editor for handling this manuscript.

**Reviewer #1:** The manuscript entitled “A novel mitovirus associated with the fungal entomopathogen Zoophthora radicans” by Adler et al. is a straightforward manuscript that reports the molecular characterization of a novel mitovirus associated to a pea aphid´s fungal entomopathogen. It is great that authors conducted the 18S amplicon sequencing and further PCR screening to unequivocally assign the host to the identified mitovirus.

Thanks to Reviewer #1 for their review of this manuscript.

**Reviewer #2:** The study by Adler et al. identifies several viruses present in metatranscriptomic sequencing data for field-collected aphids. Of these viruses, the researchers recovered a contig encoding a novel mitovirus, and its presence in aphids was associated with the co-presence of reads attributed to the fungal entomopathogen, Zoophthora radicans. The data provided is original and provides novel insights into aphid virome diversity, which also includes viruses from plants and fungi. However, there are several minor revisions regarding data availability and methods descriptions, as well major revisions regarding the phylogenetic relationships and the true host of “Zoophthora radicans mitovirus”:

Thanks to Reviewer #2 for their comments on the manuscript.

Comment #1:
- Individual aphid sample metadata should be provided either in main figures or supplemental table:

- Which samples were from winged (alate) versus unwinged (aptera) aphids?
- Which samples were aphids from T. repens versus T. pratense?
- Which individual aphid samples were put into pool 1 and pool 2 used for metatranscriptomic sequencing?

As suggested, we now include a table in the Supporting Information (S1 Table) that describes this metadata. Readers are directed to S1 Table in the following methods section sentence: “Samples were a mix of winged (alate) and unwinged (aptera) adults, metadata in S1 Table.” Host plants were identified at a genus level and to a species level, when confident.

Comment #2
- For data reproducibility purposes, the method of cDNA synthesis used for PCR screening individual aphid samples for the novel mitovirus should be stated; it is unclear what “constructed cDNA as above” (line 127) is referring to.

We added the specific cDNA synthesis kit to the referenced sentence. It now reads, “We extracted RNA from field-collected aphids and constructed cDNA via iScript cDNA Synthesis Kit by BIO-RAD, as directed by the manufacturer.”

Comment #3

- The data provided for RT-PCR confirmation of the mitovirus across the 10 aphid samples is insufficient in Figure 2. PCR gel images should be shown including expected/observed molecular weight of mitovirus amplicons as well as appropriate control groups, such as reference gene controls, cDNA synthesis reactions plus and minus reverse transcriptase, and/or mitovirus RT-PCR for the corresponding gDNA samples.

We have added a supporting figure, S2 Figure, which is gel images of the *Mitovirus* PCR screening of aphid samples used in amplicon sequencing in Figure 2.

Comment #4
- A statement of what criteria was chosen to call this a “novel” species of Mitoviridae should be included. For example, refer to the International Committee on Taxonomy of Viruses (ICTV) Mitoviridae proposal for this criteria (<https://ictv.global/taxonomy>). A phylogenetic tree comparing the novel mitovirus RdRp to several other known mitovirus RdRps would also provide a Mitoviridae genus-level assignment and provide a measure of novelty of Zoophthora radicans mitovirus compared to other mitoviruses

In the revised results we now clarify: “Species demarcation in this study was based on ICTV *Mitoviridae* Study Group (SG) criteria of less than 70% amino acid sequence identity to other viral genome sequences (Walker, et al. 2022). *Zoopthora radicans mitovirus 1* exhibits less than 70% amino acid sequence identity to the best match in the nr database (see above). We, therefore, concluded that this sequence is a newly described species of *Mitovirus* that is likely associated with *Z. radicans*, and in the NCBI submission we refer to this virus as *Zoophthora radicans mitovirus 1*.”

Comment #5
- For data reproducibility purposes, statements for the parameters used with following tools should be included, even if simply “default parameters were used”:
o STAR
o Trimmomatic
o PriceSeqFilter
o Mini-map2
o Diamond
o SPADES
o bowtie2
o Geneious Prime
o NCBI ORFfinder
o InterPro

We have added specific parameters to the bioinformatic processing methods section. The CZ ID Illumina mNGS metagenomic pipeline is publicly available on their github so we have clarified when the pipeline starts and ends in our analysis. The remaining programs (Geneious Prime, NCBI ORFfinder, InterPro) are now described with the version used and the description “with default parameters” as suggested by Reviewer #2 version. It now reads, “With completion of the CZ ID pipeline, we de novo assembled reads with homology to each putative virus using Geneious Prime version 2025.0 using default parameters for the De Novo Assemble tool. With the assembled genomes, we used the NCBI Open Reading Frame Finder (https://www.ncbi.nlm.nih.gov/orffinder/, last modified 2021-05-04) to identify protein-coding regions of the viral genome (using genetic code 1 for putative viruses, and genetic code 3 Yeast Mitochondrial for the putative mitovirus). We used interpro (https://www.ebi.ac.uk/interpro/ release 103.0) to look for conserved genes to annotate viral genomes.”

Comment #6
- The “true” host of the novel mitovirus could be established by in vitro isolation and culture of Z. radicans from a mitovirus-positive aphid, then showing that Z. radicans alone is RT-PCR positive for the mitovirus. This experiment should be performed if it’s trivial, as this would confirm the host of the novel mitovirus (otherwise it should not be called “Zoophthora radicans mitovirus”).

This would be a definitive way to associate the mitovirus to *Z.radicans*, but unfortunately, neither in vitro isolation nor culture of a *Z.radicans* from a known mitovirus-positive field collected aphid is possible. We have used the following sentence to describe the inference made from our data, “We used these data sets to infer that our newly described *Mitovirus* is likely associated with an entomopathogenic fungus called *Zoophthora radicans*”. Further, we add an additional caveat in the discussion: “We note, however, that the true host of this novel mitovirus cannot be definitely established without in vitro isolation and culturing, and caution is warranted in the use of the name *Zoophthora radicans mitovirus 1* without further investigation.”
